# Prospective Associations of Serum Adiponectin, Leptin, and Leptin-Adiponectin Ratio with Incidence of Metabolic Syndrome: The Korean Genome and Epidemiology Study

**DOI:** 10.3390/ijerph17093287

**Published:** 2020-05-08

**Authors:** Kyung Won Lee, Dayeon Shin

**Affiliations:** 1 Department of Food Science and Nutrition, Gwangju University, Gwangju 61743, Korea; kwlee@gwangju.ac.kr; 2 Department of Food and Nutrition, Inha University, Incheon 22212, Korea

**Keywords:** adiponectin, leptin, metabolic syndrome, prospective study, Koreans

## Abstract

Although the role of adiponectin and leptin in the etiology of metabolic syndrome (MetS) has been explored in various populations, limited knowledge is available on the prospective association of adiponectin and leptin with the risk of MetS development. The present study aimed to evaluate the associations of adiponectin, leptin, and the leptin-adiponectin (LA) ratio with the future risk of MetS in middle-aged and older Korean adults. Using a prospective, population-based Ansan-Ansung cohort of the Korean Genome and Epidemiology Study (KoGES), 2691 Korean adults (1317 men and 1374 women) were included in the present study. Serum adiponectin and leptin concentrations were measured using commonly available enzyme-linked immunosorbent assay kits. Multivariable Cox proportional hazard models were used to investigate the relationships of the different adiponectin and leptin concentrations and LA ratio with the incident MetS. During a mean follow-up of 6.75 years, a total of 359 (27.26%) men and 385 (28.02%) women were identified as developing new-onset MetS. After controlling for covariates, higher adiponectin levels were associated with lower incidence of MetS (hazard ratio (HR) for third vs. first tertile: 0.53, 95% confidence interval (CI): 0.40–0.70 for men and HR: 0.54, 95% CI: 0.42–0.71 for women), while higher leptin levels (HR for third vs. first tertile: 2.88, 95% CI: 2.01–4.13 for men and HR: 1.55, 95% CI: 1.13–2.13 for women) and LA ratio (HR for third vs. first tertile: 3.07, 95% CI: 2.13–4.44 for men and HR: 1.94, 95% CI: 1.41–2.66 for women) were associated with an increased incidence of MetS. Among men, in the fully adjusted models an increase by one standard deviation (SD) in adiponectin levels was associated with a 10% decrease in MetS risk (HR per SD: 0.90, 95% CI: 0.85–0.95) while leptin and LA ratio was associated with a 5% (HR per SD: 1.05, 95% CI: 1.01–1.08) and 40% (HR per SD: 1.40, 95% CI: 1.22–1.62) increase in MetS risk, respectively. Among women, a significant association with MetS risk was observed only in adiponectin levels (HR per SD: 0.91, 95% CI: 0.88–0.95). We found that higher adiponectin level was associated with a lower risk of MetS, while higher leptin level and LA ratio were associated with elevated MetS incidence, irrespective of body mass index at baseline in both Korean men and women. Adiponectin and leptin levels and LA ratio could play a role as a useful biomarker in the prediction of future MetS development among middle-aged and older Koreans.

## 1. Introduction

In contrast to three or four decades ago when infectious diseases were the main cause of death, the healthcare burden from non-communicable diseases (NCDs) continues to rise worldwide [1]. Following this trend, Korea is also encountering a rising level of NCD cases. Deaths due to NCDs account for 80% of all deaths in Korea, accounting for seven of the top 10 causes of death in the country [2]. NCDs include a variety of diseases, but one disease in particular that poses many challenges is metabolic syndrome (MetS). MetS is characterized as a cluster of metabolic conditions, including abdominal obesity, dyslipidemia, elevated fasting blood glucose, and high blood pressure [3].

According to the Metabolic Syndrome Fact Sheet in Korea 2018, one out of five Korean adults has MetS. Despite various efforts to prevent MetS, the prevalence of the disease, rising from 21.1% in 2007 to 22.4% in 2015, has not exhibited any signs of a significant decrease [4]. In addition, MetS prevalence has drastically increased for both men and women in their 50s and older. Furthermore, as MetS is known as a key risk factor for cardiovascular disease and diabetes, great emphasis is being placed on the importance of preventing and treating MetS [5,6].

As with other chronic diseases, it is necessary to identify the biomarkers associated with MetS occurrence in order to aid prevention efforts via screening of high-risk groups. Several clinically relevant biomarkers associated with MetS have been proposed, specifically adiponectin and leptin [7]. As members of the adipokine family of cytokines secreted from the adipose tissue, adiponectin and leptin carry out antagonistic functions in the body [8]. Adiponectin is involved in energy metabolism and exhibits anti-inflammatory effects, including its role as an insulin sensitizer [9]. Low adiponectin levels are found in obese individuals, and is associated with insulin resistance, diabetes, and cardiovascular disease [10,11]. Leptin, however, aids the regulation of appetite by producing a feeling of satiation when food is ingested, and maintains fat storage in the body at a consistent level [12]. Leptin is associated with pro-inflammatory effects, including regulation of the secretion of cytokines such as tumor necrosis factor-α and interleukin-6. Elevated leptin levels can also cause obesity, diabetes, and heart disease [13,14]. In addition, hyperleptinemia, rather than hyperinsulinemia or insulin resistance, has been reported as a critical risk factor for MetS [15].

Despite active research on the role of adiponectin and leptin with regards to MetS risk for diverse ethnic groups [16,17,18], investigations into the association of adiponectin and leptin for MetS risk in Korea have been limited to cross-sectional studies [19,20] or short-term follow-up studies [21,22]. Previous studies have mostly been conducted with people in the Western hemisphere, who have a different adiposity and body composition to Asian populations. Considering that the concentration of adiponectin and leptin in the blood is affected by the level of obesity [10,23], and the lower relative adiposity seen in Korean compared with Western populations, research revealing the prospective association of adiponectin and leptin to MetS risk in a Korean population, observed over an extended follow-up period, is needed with a relatively lower adiposity than Westerners, considering the effect of obesity. Therefore, the present study aimed to investigate the association of three markers—adiponectin, leptin, and the leptin-adiponectin (LA) ratio—with the risk of MetS development in middle-aged and older Korean adults, an age group experiencing a relatively drastic increase in the prevalence of MetS.

## 2. Methods

### 2.1. Data Source and Study Population

**Figure 1:**
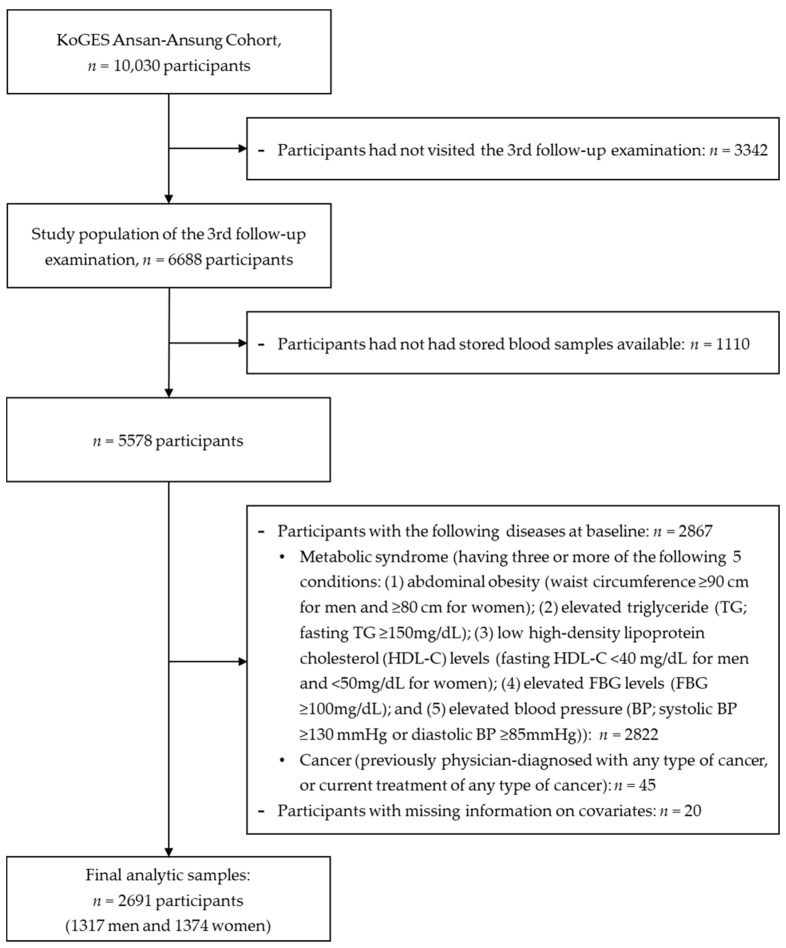
Flow diagram of the study population.

We used data from a prospective, population-based Ansan-Ansung cohort study, part of the Korean Genome and Epidemiology Study (KoGES) conducted by the Korea National Institute of Health (KNIH). In this study, 10,030 Korean adults aged 40–69 years were recruited from two Korean cities (Ansan and Ansung), and were followed up biennially over a 14-year period. Detailed study design and procedures of the Ansan-Ansung cohort study have been previously published [24]. The KNIH additionally identified 15 biomarkers, including adiponectin and leptin, using the stored blood samples obtained from individuals who participated in the third follow-up examination. Since the third follow-up examination provided information on serum adiponectin and leptin levels, we performed the analysis by considering this data as the baseline. In total, 6688 people participated in the third follow-up examination between 2007 and 2008, among whom 1100 were excluded due to an absence of stored blood samples. Of the remaining 5578 participants, those with existing metabolic syndrome (n = 2822), any type of cancer (n = 45), or missing information on covariates (n = 20) were also excluded (Figure 1). Final subjects included in the analysis comprised 2691 Korean adults (1317 men and 1374 women), followed up until 2016. The Institutional Review Board of the Korea Centers for Disease Control and Prevention reviewed and approved the KoGES study protocol. Each participant’s written informed consent was obtained in accordance with the relevant institutional guidelines and regulations. The study was approved by the Institutional Review Board of Gwangju University (IRB number: 2-1041318-A-N-01-202001-HR-001-01). The dataset of Ansan-Ansung cohort used in this study are available through online sharing service after review of the research plan by the Korea Centers for Disease Control and Prevention.

### 2.2. Measurements of Anthropometric and Biochemical Parameters

All participants visited the survey site for an interview and clinical assessments at baseline and each follow-up. Information on sociodemographic characteristics, lifestyle, medical history, anthropometric measures, and biospecimens were collected by trained staff using standardized protocols during each examination, and participants of the cohort were followed-up biennially. Height, weight, and waist circumference were measured using standard methods in light clothing and barefoot. Body mass index (BMI) was calculated based on measured height and weight (body mass (kg)/height (m^2^)). Blood pressure (BP) was measured by trained technicians using a conventional mercury sphygmomanometer (Baumanometer; Baum, Copiague, NY, USA). Systolic BP and diastolic BP were measured on both arms in the sitting position after at least 5 min of rest. The average of measurements on the left and right arms were used to define values of systolic BP and diastolic BP in this study. For the measurement of biochemical components, all blood samples were taken after fasting for 12 h. Serum adiponectin and serum leptin concentrations were measured using commonly available enzyme-linked immunosorbent assay (ELISA) kits (Mesdia, Seoul, Korea). Levels of serum adiponectin, serum leptin, and the corresponding LA ratio were analyzed as tertiles (tertile cut-offs were based on the sex-specific distributions of the main exposure variables) and continuous measures. Serum blood glucose, triglyceride (TG), high-density lipoprotein cholesterol (HDL-C) were assessed enzymatically using a Hitachi 747 chemistry analyzer (Hitachi, Tokyo, Japan). Serum high-sensitivity C-reactive protein (hs-CRP) was measured by enzymatic colorimetric methods using an automatic analyzer (ADVIA 1650 and 1800, Siemens, Tarrytown, NY, USA).

### 2.3. Ascertainment of Metabolic Syndrome

Diagnosis of MetS was carried out according to the National Cholesterol Education Program Adult Treatment Panel III (NCEP ATP III) [25] and International Diabetes Federation [26]. Based on these parameters, new-onset MetS is identified in this study by the presence of three or more of the following conditions: (1) abdominal obesity (waist circumference ≥90 cm for men and ≥80 cm for women); (2) elevated BP (systolic BP ≥ 130 mmHg or diastolic BP ≥ 85 mmHg); (3) elevated fasting blood glucose ((FBG) ≥ 100 mg/dL); (4) elevated TG (fasting TG ≥ 150 mg/dL); and (5) HDL-C levels (fasting HDL-C < 40 mg/dL for men and <50 mg/dL for women). In the survival analyses, survival time for individuals who reported any diagnosis of MetS during the follow-ups was defined as the time between the baseline and the date of the new onset of MetS. Participants who were not diagnosed with MetS until the end of the study were censored on the date of the last follow-up examination, and survival time was defined as the time between the baseline and the last follow-up.

### 2.4. Statistical Analyses

All analyses were performed for men and women separately because women had higher adiponectin and leptin levels than men. According to the statistical distribution of three indicators, we split our study population into sex-specific tertiles of serum adiponectin (cut-offs for 4.35, 5.94 μg/mL for men and 5.88, 8.09 μg/mL for women), leptin (cut-offs for 1.38, 2.91 ng/mL for men and 7.36, 12.63 ng/mL for women), and LA ratio (cut-offs for 0.263, 0.643 ng/μg for men and 0.990, 2.016 ng/μg for women). Descriptive analysis of the baseline characteristics of the study population according to tertiles of adiponectin, leptin, and LA ratio were presented as frequencies (percentages) for categorical variables and means ± standard deviations (SDs) or medians (interquartile ranges) for continuous variables. Baseline characteristics according to tertiles of three biomarkers were compared using chi-square tests, generalized linear regressions, and Kruskal–Wallis tests. We investigated the relationships between the different adiponectin and leptin concentrations and the LA ratio with incident MetS and its components to calculate hazard ratios (HRs) and 95% confidence intervals (95% CIs) using multivariable Cox proportional hazard models. Two models were applied: model 1 was adjusted for age (years); and model 2 was further adjusted for area of residence (Ansan or Ansung), education level (elementary school or lower, middle/high school, or college or higher), smoking status (never, past, or current), alcohol consumption (g/d), regular physical activity (yes or no), BMI (kg/m^2^), family history of diabetes (yes or no), and serum hs-CRP levels (mg/L). To test the linear trend, median values of each tertile group of main exposure variables were assigned to the regression models as a continuous variable. To investigate the joint effect of adiponectin and leptin concentrations on the development of MetS, individuals were cross-classified by sex-specific tertiles of serum adiponectin and leptin levels into 9 groups, and the combination of high adiponectin and low leptin levels was considered as the referent. Data management and statistical analyses were performed with SAS software version 9.4 (SAS Institute, Inc., Cary, NC, USA). A two-sided *P*-value of <0.05 was considered to be statistically significant.

## 3. Results

During a mean follow-up of 6.75 ± 2.11 years (18,162 person-years), a total of 359 (27.26%) men and 385 (28.02%) women were identified with new-onset MetS. Table 1 describes the characteristics of the study population according to LA ratio at baseline. In both men and women, individuals in the highest adiponectin tertile were more likely to be young, highly educated, and reside in rural areas (all, *P* < 0.05). Compared to those in the lowest tertile of adiponectin, those in the highest tertile had a higher average BMI, higher average leptin level and LA ratio. The prevalence of individual components of MetS differed significantly by adiponectin levels. Participants in the highest adiponectin tertile showed a higher proportion of abdominal obesity, elevated TG, and low HDL-C at baseline examination (*P* < 0.001). In addition, most of participants in the highest adiponectin tertile had at least one risk factor for MetS. Baseline characteristics of study participants according to tertiles of adiponectin and leptin are shown in Supplementary Tables S1 and S2, respectively.

Table 2 and Table 3 summarize significant associations of adiponectin, leptin, and LA ratio with 10-year MetS incidence in men and women. When models were controlled for age, area of residence, education level, smoking status, alcohol consumption, regular physical activity, BMI, family history of diabetes, and serum hs-CRP levels (model 2), higher adiponectin levels were associated with a lower incidence of MetS (HR for third vs. first tertile of adiponectin: 0.53, 95% CI: 0.40–0.70, *P*_trend_ < 0.0001 for men; 0.54, 95% CI: 0.42–0.71, *P*_trend_ < 0.0001 for women), while higher leptin levels and LA ratio were associated with increased incidence of MetS (HR for third vs. first tertile of leptin: 2.88, 95% CI: 2.01–4.13, *P*_trend_ < 0.0001 for men; 1.55, 95% CI: 1.13–2.13, *P*_trend_ < 0.0001 for women; HR for third vs. first tertile of LA ratio: 3.07, 95% CI: 2.13–4.44, *P*_trend_ < 0.0001 for men and 1.94, 95% CI: 1.41–2.66, *P*_trend_ < 0.0001 for women). Among men, in the fully adjusted models, an increase by one SD in adiponectin levels was associated with a 10% decrease in risk of MetS (HR per SD: 0.90, 95% CI: 0.85–0.95), while leptin and LA ratio was associated with a 5% (HR per SD: 1.05, 95% CI: 1.01–1.08) and 40% (HR per SD: 1.40, 95% CI: 1.22–1.62) increase in MetS risk, respectively. Among women, a significant and inverse association with MetS risk was observed only for adiponectin levels (HR per SD: 0.91, 95% CI: 0.88–0.95).

Supplementary Tables S3 and S4 show the sex-specific associations between levels of adiponectin, leptin, and the LA ratio with the incident MetS components. Higher baseline adiponectin level showed a 35% and 58% reduction in the risk of elevated TG in men and women, respectively, but an inverse association between low HDL-C and MetS incidence was found only in men. A higher leptin level at baseline was associated with an increased risk of abdominal obesity and elevated BP in both men and women. In the sex-stratified analysis, leptin level was associated with a higher incidence of elevated FBG, elevated TG, and low HDL-C in men only. Differences between the sexes regarding LA ratio and the risk of developing individual MetS components were also observed. Among men, increasing LA ratio was associated with an elevated risk of abdominal obesity, elevated FBG, and low HDL-C, while among women, LA ratio was associated with an increased risk of abdominal obesity, elevated BP, and elevated TG.

**Figure 2:**
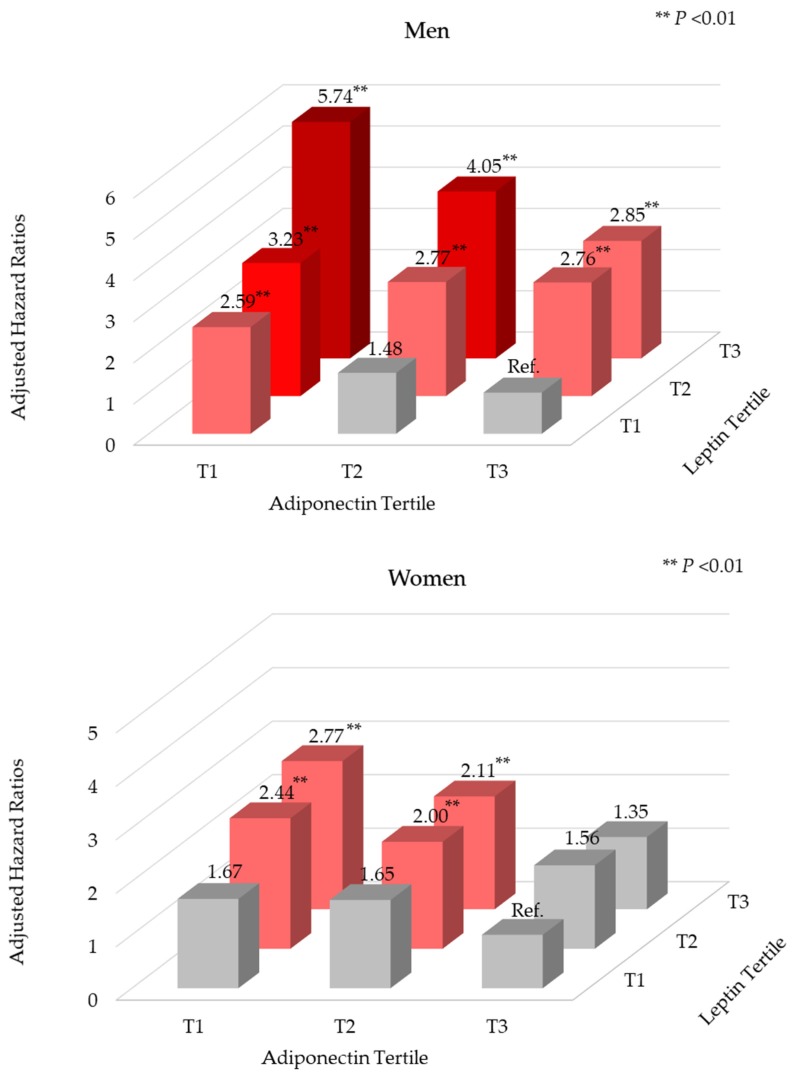
Association of metabolic syndrome by baseline adiponectin and leptin level strata in Korean adults aged 45–76 years^1^. ^1^ Model was adjusted for age (years), area of residence (Ansan or Ansung), education level (≤elementary school, middle/high school, or ≥college), smoking status (never, past, or current), alcohol consumption (g/day), regular physical activity (yes or no), BMI (kg/m^2^), family history of diabetes (yes or no), and serum hs-CRP level (mg/L).

Figure 2 shows MetS incidence in each group of cross-classified tertiles by adiponectin and leptin levels. Compared to the combination of high adiponectin-low leptin levels, the combination of low adiponectin-high leptin levels showed the greatest estimated risk among both men (HR: 5.74, 95% CI: 3.30–9.98) and women (HR: 2.77, 95% CI: 1.75–4.38). The test for interaction between tertiles of adiponectin and leptin was not statistically significant (*P*_interaction_ > 0.05).

## 4. Discussion

Middle-aged and older Koreans in the KoGES Ansan-Ansung cohort study were observed for significant relationships between serum adiponectin and leptin levels and LA ratio, and the future risk of developing MetS or its individual components. In the present study, an association between higher adiponectin levels and the lowered incidence of MetS was shown. In contrast, elevated leptin levels and a higher LA ratio were positively associated with risk of MetS development. When examining the combined effect of serum adiponectin and leptin levels by using cross-classified tertiles, the low adiponectin-high leptin combination foretold the greatest risk of developing MetS.

Adiponectin plays a critical role in insulin sensitivity and inflammation, and thus hypoadiponectinemia is thought to be linked with multiple metabolic risk factors [27]. Previous findings from epidemiological studies compare well with our results, indicating an inverse association between adiponectin levels and MetS risk. A cross-sectional analysis of a Chinese population found that those in the highest adiponectin quartile showed an increased MetS risk (odds ratio (OR): 3.38, 95% CI: 2.56–4.46) over those in the lowest quartile [28]. Another cross-sectional study of middle-aged Korean adults compared the first adiponectin quartile with those of the fourth quartile and found a decreased risk of MetS (OR: 0.32, 95% CI: 0.20–0.50 for men; OR: 0.57, 95% CI: 0.43–0.76 for women) [19]. Contrary to adiponectin, elevated leptin levels and hyperleptinemia have been demonstrated to be closely associated with metabolic abnormalities [29,30]. For example, a prospective cohort study of 748 middle-aged white adults reported that baseline leptin levels accurately predicted the future risk of developing MetS and obesity [31]. In overweight and obese European adolescents, increased leptin level was associated with an increased prevalence of MetS [32]. Adiponectin and leptin are produced primarily by adipose tissue [8,33] and are related to measures of obesity, such as BMI [34]. It has been seen that adiponectin levels are lowered and leptin levels are elevated in those with obesity [35,36]. Previous studies revealed that serum adiponectin and leptin levels were significant risk factors for MetS in a Western population with high adiposity. We extend these findings to a Korean population, with a relatively lower adiposity compared to the Western population.

We found that LA ratio was a more accurate marker than adiponectin or leptin alone for identification of individuals at risk for MetS in the Korean adult population. In parallel with our findings, previous investigations have reported the utility of LA ratio in the early diagnosis of MetS. A study involving 2046 Chinese men and women aged 60–96 years indicated that one SD increase of LA ratio was associated with an 83% (OR: 1.83, 95% CI: 1.38–2.45, *P* < 0.001) and 132% (OR: 2.32, 95% CI: 1.82–2.96, *P* < 0.001) higher risk of MetS in men and women, respectively [17]. In addition, receiver operating characteristic curve analysis confirmed that the use of LA ratio significantly improved diagnostic ability by 9.6% in men and 7.7% in women compared to adiponectin alone. Another study based on a French population found that LA ratio was positively correlated with the number of MetS cases, and with each component thereof [37]. Mirza et al. also reported that adiponectin-leptin ratio had a greater diagnostic ability for MetS compared with either adiponectin or leptin alone [38]. Our findings suggest that adiponectin, leptin, and LA ratio might be promising biomarkers for predicting the future risk of MetS in the middle-aged and older Korean adults, particularly in those with one or more MetS risk factors. The importance of early detection of diseases has been recognized in prevention and timely management. Investigations have been made to identify useful biomarkers that enable early diagnosis of diseases [39,40,41]. Adiponectin and leptin levels are known to be significantly associated with obesity. In the current study, we found that adiponectin, leptin, and LA ratio were prospectively associated with MetS risk among Korean adults even after adjusting for obesity, which is one of the most significant risk factors for MetS. Therefore, monitoring adiponectin and leptin in combination with the measurement of obesity may provide a simple, easy, and cost-effective approach for early detection of MetS.

In this study, adiponectin, leptin, and LA ratio were differentially associated with the risk of MetS development by sex. Although the reason for the sex difference in these associations has not been clearly elucidated, sexual dimorphism could provide a partial explanation. First, sex differences in adiposity and fat distribution via sex hormones such as androgen and estrogen could be one reason. In general, women have twice as much body fat as men, and different body fat distributions are observed between men and women. Body fat in women is mainly distributed in the gluteal-femoral area such as the hips and thighs, whereas men accumulate body fat in the abdominal or visceral area [42]. Adiponectin and leptin gene expression is more likely to occur in subcutaneous than in visceral adipose tissue [43,44]. Furthermore, serum leptin levels are affected only by leptin produced by subcutaneous adipose tissue [45]. This may cause the differences in levels of adiponectin and leptin seen in the blood of men and women. Second, another possible explanation is a sex difference of genetic variants of adipokine genes. Christen et al. [46] reported that a single nucleotide polymorphism near the LEP gene (rs791595) altered serum leptin levels. Carrying one LEP risk allele was associated with a 3 μg/L increase in leptin in women, but such an association was not observed in men. Lastly, metabolic flexibility could be a key explanation in sex differences observed in the relationships between adiponectin, leptin, and LA ratio on incident MetS. Metabolic flexibility is the ability of the human body to adapt to metabolic changes. Additionally, metabolic flexibility may be determined by how closely the transition between fat oxidation and glucose oxidation occurs to meet metabolic requirements [47,48]. Female skeletal muscle is optimized for oxidation and lipid storage, thus women have a greater capacity for glucose and fatty acid utilization [49]. Furthermore, it has been documented that serum adiponectin levels correlate with metabolic flexibility [50]. Adiponectin, which exists at a higher level in women than in men, may promote metabolic flexibility by increasing lipid synthesis and storage under fed conditions and enhancing lipid catabolism under fasting conditions [51]. Together, these suggest that women are more metabolically flexible than men in a metabolically challenged state.

To the best of our knowledge, this is the first prospective study investigating the associations of serum adiponectin, leptin, and LA ratio with MetS incidence. The present study has several strengths, including the population-based prospective design without exposure to recall bias, the large sample size, length of follow-up period, and controls for possible covariates that could affect the relationships between adiponectin and leptin levels, and MetS (e.g., sex, BMI, and hs-CRP concentration). However, there were also limitations that need to be mentioned. First, we estimated the associations between the three markers and MetS incidence based on a single measurement of adiponectin and leptin levels due to lack of available data. The results of this study do not provide a comprehensive explanation for the effect of alterations in adiponectin and leptin levels over time on future MetS risk. Second, we used only total adiponectin concentration. It has been well documented that high-molecular-weight adiponectin is the most active fraction of adiponectin [52] and contributes widely to insulin-sensitizing activity [53]. However, since previous epidemiological studies reported that total adiponectin and high-molecular-weight adiponectin showed a similar predictive ability in insulin resistance and metabolic abnormalities [54,55], the measurement of total adiponectin used in this study was considered sufficient. Third, baseline dietary information was not included in the statistical models due to unavailability. Nevertheless, we included alcohol consumption, which is one of the major dietary factors contributing to the serum levels of adiponectin and leptin [56,57,58]. Further investigation exploring interactions between diet and adiponectin and leptin levels over time on MetS risk is needed. Fourth, although we adjusted for various potential confounders related to exposure and outcome variables of interest in this study, the issue of unmeasured or unidentified confounders still remains. Moreover, our study participants consist of middle-aged and older Korean adults residing in specific areas (two cities in Gyeonggi province) of Korea. Our findings may be difficult to generalize to other Korean populations with different ages or lifestyle characteristics. Thus, additional investigations are needed to confirm the applicability of our results to the general Korean population.

## 5. Conclusions

In conclusion, higher adiponectin levels were associated with a lower risk of developing MetS, while higher leptin levels and LA ratio were associated with an elevated MetS incidence in both men and women. Our results support the role of adiponectin and leptin levels and LA ratio as useful predictive markers to measure the risk of future MetS development among middle-aged and older Koreans. Continuous monitoring of adiponectin, leptin, and LA ratio could help detect high-risk individuals for MetS. Furthermore, the present results warrant further investigation into how distinct trajectory patterns of serum adiponectin and leptin levels are associated with the risk of incident MetS in a large population-based prospective study with a longer follow-up period.

## Figures and Tables

**Table 1:** Characteristics of the study participants according to leptin-adiponectin ratios in Korean men and women aged 45–76 years.

	Men				Women			
	Tertile of LA Ratio^1^				Tertile of LA Ratio			
	T1 (Lowest)	T2	T3 (Highest)	*P* ^3^	T1 (Lowest)	T2	T3 (Highest)	*P*
	(n = 439)	(n = 439)	(n = 439)		(n = 458)	(n = 458)	(n = 458)	
Age (years)	59.07 ± 8.53^2^	56.63 ± 8.51	54.53 ± 7.68	<0.0001	56.84 ± 8.62	53.86 ± 6.95	53.06 ± 6.70	<0.0001
Area of residence				<0.0001				<0.0001
Ansan	245 (55.81)	180 (41.00)	121 (27.56)		230 (50.22)	162 (35.37)	124 (27.07)	
Ansung	194 (44.19)	259 (59.00)	318 (72.44)		228 (49.78)	296 (64.63)	334 (72.93)	
Education level				<0.0001				<0.0001
≤elementary school	102 (23.23)	82 (18.68)	38 (8.66)		157 (34.28)	122 (26.64)	98 (21.40)	
middle/high school	260 (59.23)	254 (57.86)	265 (60.36)		266 (58.08)	292 (63.76)	311 (67.90)	
≥college	77 (17.54)	103 (23.46)	136 (30.98)		35 (7.64)	44 (9.61)	49 (10.70)	
Smoking status				0.0688				0.4408
Never	114 (25.97)	134 (30.52)	114 (25.97)		452 (98.69)	451 (98.47)	449 (98.03)	
Past	151 (34.40)	170 (38.72)	193 (43.96)		2 (0.44)	3 (0.66)	3 (0.66)	
Current	174 (39.64)	135 (30.75)	132 (30.07)		4 (0.87)	4 (0.87)	6 (1.31)	
Alcohol consumption (grams/day)^4^	4.70 (0.00–18.81)	4.70 (0.00–20.26)	10.13 (0.60–25.25)	0.0020	0.00 (0.00–0.26)	0.00 (0.00–0.87)	0.00 (0.00–0.31)	0.1584
Regular physical activity				0.006				0.1405
Yes	161 (36.67)	174 (39.64)	201 (45.79)		171 (37.34)	208 (45.41)	193 (42.14)	
No	278 (63.33)	265 (60.36)	238 (54.21)		287 (62.66)	250 (54.59)	265 (57.86)	
Family history of diabetes				0.0535				0.6564
Yes	3 (0.68)	7 (1.59)	10 (2.28)		11 (2.40)	7 (1.53)	13 (2.84)	
No	436 (99.32)	432 (98.41)	429 (97.72)		447 (97.60)	451 (98.47)	445 (97.16)	
Body mass index (kg/m^2^)	21.16 ± 2.04	23.20 ± 1.87	24.83 ± 2.07	<0.0001	21.70 ± 2.11	23.53 ± 2.04	25.19 ± 2.41	<0.0001
hs-CRP (mg/dL)^4^	0.49 (0.27–1.19)	0.55 (0.30–1.12)	0.86 (0.44–1.68)	<0.0001	0.35 (0.22–0.64)	0.43 (0.28–0.77)	0.65 (0.37–1.22)	<0.0001
Adiponectin (μg/mL)^4^	6.52 (5.37–7.91)	5.03 (4.06–6.15)	4.00 (3.17–4.84)	<0.0001	8.74 (7.36–10.93)	6.87 (5.66–8.22)	5.21 (4.10–6.51)	<0.0001
Leptin (ng/mL)^4^	1.01 (1.01–1.11)	2.15 (1.64–2.70)	4.15 (3.21–5.76)	<0.0001	5.18 (3.49–6.67)	9.93 (8.16–12.06)	16.35 (13.15–21.10)	<0.0001
LA ratio (ng/μg)^4^	0.17 (0.14–0.21)	0.43 (0.34–0.52)	1.01 (0.81–1.41)	<0.0001	0.63 (0.40–0.80)	1.46 (1.20–1.74)	3.00 (2.46–3.99)	<0.0001
MetS components, n (%)								
Abdominal obesity	8 (1.82)	39 (8.88)	84 (19.13)	<0.0001	31 (6.77)	55 (12.01)	94 (20.52)	<0.0001
Elevated BP	97 (22.10)	126 (28.70)	108 (24.60)	0.3923	108 (23.58)	71 (15.50)	77 (16.81)	0.0085
Elevated FBG	58 (13.21)	77 (17.54)	100 (22.78)	0.0002	19 (4.15)	20 (4.37)	31 (6.77)	0.0715
Elevated TG	35 (7.97)	77 (17.54)	125 (28.47)	<0.0001	17 (3.71)	47 (10.26)	73 (15.94)	<0.0001
Low HDL-C	92 (20.96)	125 (28.47)	134 (30.52)	0.0014	207 (45.20)	252 (55.02)	272 (59.39)	<0.0001
No. of MetS components at baseline				<0.0001				<0.0001
0	210 (47.84)	118 (26.88)	73 (16.63)		165 (36.03)	127 (27.73)	85 (18.56)	
1	168 (38.27)	198 (45.10)	181 (41.23)		204 (44.54)	217 (47.38)	199 (43.45)	
2	61 (13.90)	123 (28.02)	185 (42.14)		89 (19.43)	114 (24.89)	174 (37.99)	

LA ratio, leptin-adiponectin ratio; T, tertile; hs-CRP, high-sensitivity C-reactive protein; MetS, metabolic syndrome; BP, blood pressure; FBG, fasting blood glucose; TG, triglyceride; HDL-C, high-density lipoprotein cholesterol. ^1^ Cut-offs for tertiles 1–3 of LA ratio as follows: <0.263, 0.263–0.643, and >0.643 ng/μg in men and <0.990, 0.990–2.016, and >2.016 ng/μg in women, respectively. ^2^ Values are number (percentage) for categorical variables and mean ± standard deviation for continuous variables. ^3^ *P* values based on chi-square tests for categorical variables and generalized linear regressions for continuous variables. ^4^ Values are presented as the median (lower quartile-upper quartile), and the *P* values were from the Kruskal–Wallis tests.

**Table 2:** Adjusted hazard ratios (with 95% confidence intervals) for metabolic syndrome according to baseline serum adiponectin, leptin levels, and leptin-adiponectin ratio in Korean men aged 45–76 years.

	Tertile			*P*_trend_^2^	Per 1 SD Increment	*P*
	T1 (Lowest)	T2	T3 (Highest)			
	HR (95% CI)	HR (95% CI)	HR (95% CI)		HR (95% CI)	
Adiponectin						
Person-years	2748	2947	3075		8770	
Incident cases (*n*)	167	113	79		359	
Rate per 1000person-years	60.8	38.3	25.7		40.9	
Median	3.5	5.1	7.3		5.1	
Ranges	0.48–4.34	4.36–5.93	5.94–25.04		0.48–25.04	
Model 1 ^1^	1.00	0.62 (0.49–0.79)	0.39 (0.30–0.52)	<0.0001	0.85 (0.80–0.90)	<0.0001
Model 2	1.00	0.71 (0.56–0.91)	0.53 (0.40–0.70)	<0.0001	0.90 (0.85–0.95)	0.0003
Leptin						
Person-years	3145	2951	2674		8770	
Incident cases (*n*)	55	119	185		359	
Rate per 1000 person-years	17.5	40.3	69.2		40.9	
Median	1.0	2.1	4.3		2.1	
Ranges	1.01–1.37	1.38–2.91	2.91–24.08		1.01–24.08	
Model 1	1.00	2.39 (1.73–3.29)	4.11 (3.04–5.57)	<0.0001	1.14 (1.11–1.17)	<0.0001
Model 2	1.00	1.95 (1.39–2.74)	2.88 (2.01–4.13)	<0.0001	1.05 (1.01–1.08)	0.0176
LA ratio						
Person-years	3150	2953	2667		8770	
Incident cases (*n*)	51	124	184		359	
Rate per 1000 person-years	16.2	42.0	69.0		40.9	
Median	0.2	0.4	1.0		0.4	
Ranges	0.1–0.3	0.3–0.6	0.6–5.3		0.1–5.3	
Model 1	1.00	2.72 (1.96–3.77)	4.66 (3.39–6.39)	<0.0001	1.74 (1.56–1.94)	<0.0001
Model 2	1.00	2.16 (1.53–3.03)	3.07 (2.13–4.44)	<0.0001	1.40 (1.22–1.62)	<0.0001

T, tertile; HR, hazard ratio; CI, confidence interval; LA ratio, leptin-adiponectin ratio; BMI, body mass index; hs-CRP, high-sensitivity C-reactive protein. ^1^ Model 1 was adjusted for age (years); model 2 was additionally adjusted for area of residence (Ansan or Ansung), education level (≤elementary school, middle/high school, or ≥college), smoking status (never, past, or current), alcohol consumption (g/day), regular physical activity (yes or no), BMI (kg/m^2^), family history of diabetes (yes or no), and serum hs-CRP level (mg/L). ^2^ Tests for trend linearity were conducted with the Wald test by considering the median values of each tertile of adiponectin, leptin, and LA ratio as continuous variables in the analytical models.

**Table 3:** Adjusted hazard ratios (with 95% confidence intervals) for metabolic syndrome according to baseline serum adiponectin, leptin levels, and leptin-adiponectin ratio in Korean women aged 45–76 years.

	Tertile			*P*_trend_^2^	Per 1 SD Increment	*P*
	T1 (Lowest)	T2	T3 (Highest)			
	HR (95% CI)	HR (95% CI)	HR (95% CI)		HR (95% CI)	
Adiponectin						
Person-years	3069	3132	3191		9392	
Incident cases (*n*)	154	131	100		385	
Rate per 1000 person-years	50.2	41.8	31.3		41.0	
Median	4.7	6.9	9.6		6.9	
Ranges	0.87–5.88	5.88–8.09	8.10–27.78		0.9–27.8	
Model 1 ^1^	1.00	0.81 (0.64–1.02)	0.51 (0.39–0.66)	<0.0001	0.90 (0.86–0.93)	<0.0001
Model 2	1.00	0.82 (0.65–1.03)	0.54 (0.42–0.71)	<0.0001	0.91 (0.88–0.95)	<0.0001
Leptin						
Person-years	3296	3145	2951		9392	
Incident cases (*n*)	86	128	171		385	
Rate per 1000 person-years	26.1	40.7	57.9		41.0	
Median	5.1	9.7	17.1		9.7	
Ranges	1.01–7.33	7.37–12.62	12.65–52.36		1.0–52.4	
Model 1	1.00	1.79 (1.36–2.36)	2.47 (1.90–3.21)	<0.0001	1.04 (1.03–1.05)	<0.0001
Model 2	1.00	1.45 (1.09–1.94)	1.55 (1.13–2.13)	<0.0001	1.01 (0.99–1.02)	0.4084
LA ratio						
Person-years	3273	3169	2950		9392	
Incident cases (*n*)	88	120	177		385	
Rate per 1000 person-years	26.9	37.9	60.0		41.0	
Median	5.1	9.7	17.1		1.5	
Ranges	1.0–7.3	7.4–12.6	12.6–52.4		0.1–24.6	
Model 1	1.00	1.67 (1.26–2.20)	2.78 (2.14–3.62)	<0.0001	1.11 (1.08–1.15)	<0.0001
Model 2	1.00	1.37 (1.02–1.84)	1.94 (1.41–2.66)	<0.0001	1.04 (0.99–1.09)	0.1096

T, tertile; HR, hazard ratio; CI, confidence interval; LA ratio, leptin-adiponectin ratio; BMI, body mass index; hs-CRP, high-sensitivity C-reactive protein. ^1^ Model 1 was adjusted for age (years); model 2 was additionally adjusted for area of residence (Ansan or Ansung), education level (≤elementary school, middle/high school, or ≥college), smoking status (never, past, or current), alcohol consumption (g/day), regular physical activity (yes or no), BMI (kg/m^2^), family history of diabetes (yes or no), and serum hs-CRP level (mg/L). ^2^ Tests for trend linearity were conducted with the Wald test by considering the median values of each tertile of adiponectin, leptin, and LA ratio as continuous variables in the analytical models.

## Supplementary Materials

The following are available online at https://www.mdpi.com/1660-4601/17/9/3287/s1, Table S1: Characteristics of the study participants according to adiponectin levels in Korean men and women aged 45–76 years; Table S2: Characteristics of the study participants according to leptin levels in Korean men and women aged 45–76 years; Table S3: Adjusted hazard ratios (with 95% confidence intervals) for each component of metabolic syndrome according to baseline serum adiponectin, leptin levels, and leptin-adiponectin ratio in Korean men aged 45–76 years; Table S4: Adjusted hazard ratios (with 95% confidence intervals) for each component of metabolic syndrome according to baseline serum adiponectin, leptin levels, and leptin-adiponectin ratio in Korean women aged 45–76 years.
