# Supplementary material for: Prospective Associations of Serum Adiponectin, Leptin, and Leptin-Adiponectin Ratio with Incidence of Metabolic Syndrome: The Korean Genome and Epidemiology Study
Source: Int J Environ Res Public Health. 2020 May 8;17(9):3287. doi: 10.3390/ijerph17093287 (PMC7246697; doi:10.3390/ijerph17093287)
Supplement: Supplementary file 1 [file ijerph-17-03287-s001.pdf]

Supplementary Table S1. Characteristics of the study participants according to adiponectin levels in Korean men and women aged 45–76 years.

	Men				Women			
	Tertile of Adiponectin[1]				Tertile of Adiponectin			
	**T1 (Lowest) (n = 439)**	**T2 (n = 439)**	**T3 (Highest) (n = 439)**	***P*[3]**	**T1 (Lowest) (n = 458)**	**T2 (n = 458)**	**T3 (Highest) (n = 458)**	***P***
Age (years)	54.33 ± 7.27[2]	56.33 ± 8.29	59.57 ± 8.89	<0.0001	52.96 ± 6.91	53.91 ± 7.06	56.90 ± 8.34	<0.0001
Area of residence				<0.0001				<0.0001
Ansan	153 (34.85)	169 (38.50)	224 (51.03)		138 (30.13)	161 (35.15)	217 (47.38)	
Ansung	286 (65.15)	270 (61.50)	215 (48.97)		320 (69.87)	297 (64.85)	241 (52.62)	
Education level				<0.0001				<0.0001
≤elementary school	44 (10.02)	75 (17.08)	103 (23.46)		101 (22.05)	107 (23.36)	169 (36.90)	
middle/high school	280 (63.78)	254 (57.86)	779 (55.81)		302 (65.94)	308 (67.25)	259 (56.55)	
≥college	115 (26.20)	110 (25.06)	316 (20.73)		55 (12.01)	43 (9.39)	30 (6.55)	
Smoking status				0.0271				0.0450
Never	107 (24.37)	124 (28.25)	131 (29.84)		446 (97.38)	453 (98.91)	453 (98.91)	
Past	162 (36.90)	187 (42.60)	165 (27.59)		3 (0.66)	3 (0.66)	2 (0.44)	
Current	170 (38.72)	128 (29.16)	143 (32.57)		9 (1.97)	2 (0.44)	3 (0.66)	
Alcohol consumption (grams/day)	10.13 (0.95–26.05)	5.79 (0.00–22.07)	3.80 (0.00–18.81)	<0.0001	0.00 (0.00–0.58)	0.00 (0.00–0.57)	0.00 (0.00–0.29)	0.4771
Regular physical activity				0.0993				0.0815
Yes	192 (43.74)	176 (40.09)	168 (38.27)		194 (42.36)	210 (45.85)	168 (36.68)	
No	247 (56.26)	263 (59.91)	271 (61.73)		264 (57.64)	248 (54.15)	290 (63.32	
Family history of diabetes				0.0024				0.0752
Yes	12 (2.73)	7 (1.59)	1 (0.23)		15 (3.28)	9 (1.97)	7 (1.53)	
No	427 (97.27)	432 (98.41)	438 (99.77)		443 (96.72)	449 (98.03)	451 (98.47)	
Body mass index (kg/m²)	1.66 ± 3.20	1.75 ± 5.64	1.47 ± 4.15	0.6259	1.49 ± 7.02	1.02 ± 2.20	1.07 ± 3.99	0.2681
hs-CRP (mg/dL)[4]	0.73 (0.39–1.57)	0.61 (0.32–1.38)	0.51 (0.28–1.15)	<0.0001	0.60 (0.32–1.15)	0.43 (0.26–0.83)	0.39 (0.24–0.72)	<0.0001
Adiponectin (μg/mL)[4]	3.51 (2.98–3.99)	5.08 (4.76–5.50)	7.28 (6.50–8.29)	<0.0001	4.70 (3.97–5.30)	6.87 (6.34–7.49)	9.64 (8.69–11.25)	<0.0001
Leptin (ng/mL)[4]	2.64 (1.61–3.93)	2.26 (1.07–3.73)	1.39 (1.01–2.69)	<0.0001	11.41 (7.76–16.3)	10.01 (6.64–14.86)	7.65 (4.90–12.03)	<0.0001
LA ratio (ng/μg)[4]	0.80 (0.48–1.20)	0.44 (0.22–0.73)	0.18 (0.14–0.36)	<0.0001	2.58 (1.66–3.87)	1.46 (0.94–2.17)	0.76 (0.46–1.23)	<0.0001
MetS components, *n* (%)								
Abdominal obesity	46 (10.48)	52 (11.85)	33 (7.52)	0.1428	63 (13.76)	51 (11.14)	66 (14.41)	0.7690
Elevated BP	105 (23.92)	118 (26.88)	108 (24.60)	0.8155	64 (13.97)	83 (18.12)	109 (23.8)	0.0001
Elevated FBG	93 (21.18)	68 (15.49)	74 (16.86)	0.0941	33 (7.21)	23 (5.02)	14 (3.06)	0.0043
Elevated TG	129 (29.38)	73 (16.63)	35 (7.97)	<0.0001	78 (17.03)	37 (8.08)	22 (4.8)	<0.0001
Low HDL-C	151 (34.40)	119 (27.11)	81 (18.45)	<0.0001	308 (67.25)	232 (50.66)	191 (41.7)	<0.0001

No. of MetS components at baseline				<0.0001				<0.0001
0	84 (19.13)	132 (30.07)	185 (42.14)		78 (17.03)	137 (29.91)	162 (35.37)	
1	186 (42.37)	184 (41.91)	177 (40.32)		214 (46.72)	216 (47.16)	190 (41.48)	
2	169 (38.50)	123 (28.02)	77 (17.54)		166 (36.24)	105 (22.93)	106 (23.14)	

T, tertile; hs-CRP, high-sensitivity C-reactive protein; LA ratio, leptin-adiponectin ratio; MetS, metabolic syndrome; BP, blood pressure; FBG, fasting blood glucose; TG, triglyceride; HDL-C, high-density lipoprotein cholesterol. [1] Cut-offs for tertiles 1–3 of adiponectin levels as follows: <4.35, 4.35–5.94, and >5.94 μg/mL in men and <5.88, 5.88–8.09, and >8.09 μg/mL in women, respectively. [2] Values are number (percentage) for categorical variables and mean ± standard deviation for continuous variables. [3] P values based on chi-square tests for categorical variables and generalized linear regressions for continuous variables. [4] Values are presented as the median (lower quartile-upper quartile), and the P values were from the Kruskal–Wallis tests.

Supplementary Table S2. Characteristics of the study participants according to leptin levels in Korean men and women aged 45–76 years.

	Men				Women			
	Tertile of Leptin[1]				Tertile of Leptin			
	**T1 (Lowest) (n = 439)**	**T2 (n = 439)**	**T3 (Highest) (n = 439)**	***P*[3]**	**T1 (Lowest) (n = 458)**	**T2 (n = 458)**	**T3 (Highest) (n = 458)**	***P***
Age (years)	58.35 ± 8.47[2)]	56.35 ± 8.38	55.53 ± 8.28	<0.0001	59.07 ± 8.53	56.63 ± 8.51	54.53 ± 7.68	<0.0001
Area of residence				<0.0001				<0.0001
Ansan	253 (57.63)	168 (38.27)	125 (28.47)		245 (55.81)	180 (41.00)	121 (27.56)	
Ansung	186 (42.37)	271 (61.73)	314 (71.53)		194 (44.19)	259 (59.00)	318 (72.44)	
Education level				<0.0001				<0.0001
≤elementary school	102 (23.23)	68 (15.49)	52 (11.85)		102 (23.23)	82 (18.68)	38 (8.66)	
middle/high school	255 (58.09)	274 (62.41)	250 (56.95)		260 (59.23)	254 (57.86)	265 (60.36)	
≥college	82 (18.68)	97 (22.10)	137 (31.21)		77 (17.54)	103 (23.46)	136 (30.98)	
Smoking status				0.0001				0.0688
Never	105 (23.92)	133 (30.30)	124 (28.25)		114 (25.97)	134 (30.52)	114 (25.97)	
Past	153 (34.85)	158 (35.99)	203 (46.24)		151 (34.40)	170 (38.72)	193 (43.96)	
Current	181 (41.23)	148 (33.71)	112 (25.51)		174 (39.64)	135 (30.75)	132 (30.07)	
Alcohol consumption (grams/day)	4.70 (0.00–18.81)	7.52 (0.00–22.43)	7.52 (0.00–24.02)	0.1026	0.00 (0.00–0.29)	0.00 (0.00–0.62)	0.00 (0.00–0.00)	0.2697
Regular physical activity				0.0111				0.006
Yes	157 (35.76)	185 (42.14)	194 (44.19)		161 (36.67)	174 (39.64)	201 (45.79)	
No	282 (64.24)	254 (57.86)	245 (55.81)		278 (63.33)	265 (60.36)	238 (54.21)	
Family history of diabetes				0.5811				0.0535
Yes	5 (1.14)	8 (1.82)	7 (1.59)		3 (0.68)	7 (1.59)	10 (2.28)	
No	434 (98.86)	431 (98.18)	432 (98.41)		436 (99.32)	432 (98.41)	429 (97.72)	
Body mass index (kg/m^2)	21.10 ± 2.07	23.31 ± 1.77	24.78 ± 2.11	<0.0001	21.16 ± 2.04	23.20 ± 1.87	24.83 ± 2.07	<0.0001

hs-CRP (mg/dL)[4]	0.50 (0.28–1.20)	0.56 (0.32–1.19)	0.79 (0.42–1.59)	<0.0001	0.35 (0.22–0.65)	0.43 (0.26–0.80)	0.62 (0.37–1.16)	<0.0001
Adiponectin (μg/mL)[4]	5.93 (4.79–7.46)	4.91 (3.70–6.22)	4.58 (3.78–5.78)	<0.0001	7.93 (6.04–9.92)	6.69 (5.26–8.40)	6.20 (4.80–7.91)	<0.0001
Leptin (ng/mL)[4]	1.01 (1.01–1.01)	2.10 (1.72–2.50)	2.99 (3.50–5.76)	<0.0001	5.13 (3.48–6.23)	9.74 (8.47–11.12)	17.12 (14.52–21.23)	<0.0001
LA ratio (ng/μg)[4]	0.18 (0.14–0.22)	0.43 (0.31–0.58)	0.98 (0.72–1.40)	<0.0001	0.65 (0.40–0.85)	1.47 (1.10–1.86)	2.80 (2.14–3.97)	<0.0001
MetS components, n (%)								
Abdominal obesity	7 (1.59)	31 (7.06)	93 (21.18)	<0.0001	23 (5.02)	54 (11.79)	103 (22.49)	<0.0001
Elevated BP	93 (21.18)	125 (28.47)	113 (25.74)	0.1198	94 (20.52)	76 (16.59)	86 (18.78)	0.4974
Elevated FBG	59 (13.44)	81 (18.45)	95 (21.64)	0.0015	17 (3.71)	24 (5.24)	29 (6.33)	0.0715
Elevated TG	46 (10.48)	76 (17.31)	115 (26.20)	<0.0001	21 (4.59)	48 (10.48)	68 (14.85)	<0.0001
Low HDL-C	101 (23.01)	125 (28.47)	125 (28.47)	0.0671	226 (49.34)	270 (58.95)	235 (51.31)	0.5513
No. of MetS components at baseline				<0.0001				<0.0001
0	200 (45.56)	126 (28.70)	75 (17.08)		163 (35.59)	111 (24.24)	103 (22.49)	
1	172 (39.18)	188 (42.82)	187 (42.60)		209 (45.63)	222 (48.47)	189 (41.27)	
2	67 (15.26)	125 (28.47)	177 (40.32)		86 (18.78)	125 (27.29)	166 (36.24)	

T, tertile; hs-CRP, high-sensitivity C-reactive protein; LA ratio, leptin-adiponectin ratio; MetS, metabolic syndrome; BP, blood pressure; FBG, fasting blood glucose; TG, triglyceride; HDL-C, high-density lipoprotein cholesterol. [1] Cut-offs for tertiles 1-3 of leptin levels as follows: <1.38, 1.38–2.91, and >2.91 ng/mL in men and <7.36, 7.36–12.63, and >12.63 ng/mL in women, respectively. [2] Values are number (percentage) for categorical variables and mean ± standard deviation for continuous variables. [3] P values based on chi-square tests for categorical variables and generalized linear regressions for continuous variables. [4] Values are presented as the median (lower quartile-upper quartile), and the P values were from the Kruskal–Wallis tests.

Supplementary Table S3. Adjusted hazard ratios (with 95% confidence intervals) for each component of metabolic syndrome according to baseline serum adiponectin, leptin levels, and leptin-adiponectin ratio in Korean men aged 45–76 years.[1]

MetS Components	Tertile			P_{trend}[2]
	**T1 (Lowest)**	**T2**	**T3 (Highest)**	
	**HR (95% CI)**	**HR (95% CI)**	**HR (95% CI)**	
		Adiponectin		
Abdominal obesity	1.00	0.69 (0.47–1.02)[1]	0.91 (0.61–1.37)	<0.0001
Elevated BP	1.00	1.24 (0.94–1.65)	0.94 (0.68–1.30)	0.0002
Elevated FBG	1.00	1.00 (0.74–1.35)	0.88 (0.63–1.22)	0.0007
Elevated TG	1.00	0.67 (0.45–0.99)	0.65 (0.44–0.96)	0.0725
Low HDL-C	1.00	0.64 (0.45–0.92)	0.53 (0.36–0.78)	0.0035
		Leptin		
Abdominal obesity	1.00	1.42 (0.84–2.38)	2.65 (1.56–4.51)	<0.0001
Elevated BP	1.00	1.22 (0.88–1.70)	1.56 (1.08–2.25)	0.0001
Elevated FBG	1.00	1.55 (1.09–2.20)	1.86 (1.26–2.76)	<0.0001
Elevated TG	1.00	1.68 (1.10–2.58)	2.43 (1.47–4.02)	<0.0001
Low HDL-C	1.00	2.28 (1.47–3.56)	2.59 (1.58–4.25)	<0.0001
		LA ratio		
Abdominal obesity	1.00	1.67 (1.00–2.81)	2.40 (1.40–4.10)	0.0046
Elevated BP	1.00	1.35 (0.97–1.86)	1.32 (0.90–1.94)	0.1921
Elevated FBG	1.00	1.33 (0.95–1.87)	1.63 (1.10–2.41)	0.0491
Elevated TG	1.00	1.59 (1.07–2.36)	1.61 (0.97–2.67)	0.0620
Low HDL-C	1.00	2.03 (1.32–3.11)	2.63 (1.60–4.32)	0.0005

MetS, metabolic syndrome; T, tertile; HR, hazard ratio; CI, confidence interval; BP, blood pressure; FBG, fasting blood glucose; TG, triglyceride; HDL-C, high-density lipoprotein cholesterol, LA ratio, leptin-adiponectin ratio; BMI, body mass index; hs-CRP, high-sensitivity C-reactive protein. [1] Model was adjusted for age (years), area of residence (Ansan or Ansung), education level (≤elementary school, middle/high school, or ≥college), smoking status (never, past, or current), alcohol consumption (g/day), regular physical activity (yes or no), BMI (kg/m^2), family history of diabetes (yes or no), and serum hs-CRP level (mg/L). [2] Tests for trend linearity were conducted with the Wald test by considering the median values of each tertile of adiponectin, leptin, and LA ratio as continuous variables in the analytical models.

Supplementary Table S4. Adjusted hazard ratios (with 95% confidence intervals) for each component of metabolic syndrome according to baseline serum adiponectin, leptin levels, and leptin-adiponectin ratio in Korean women aged 45–76 years.[1]

MetS Components	Tertile			P_{trend}[2]
	**T1 (Lowest)**	**T2**	**T3 (Highest)**	
	**HR (95% CI)**	**HR (95% CI)**	**HR (95% CI)**	
		Adiponectin		
Abdominal obesity	1.00	0.93 (0.70–1.23)	0.83 (0.61–1.13)	0.0002
Elevated BP	1.00	1.10 (0.82–1.48)	0.81 (0.59–1.11)	<0.0001
Elevated FBG	1.00	0.99 (0.72–1.35)	0.76 (0.54–1.07)	<0.0001
Elevated TG	1.00	0.75 (0.57–0.99)	0.42 (0.30–0.58)	0.0005
Low HDL-C	1.00	0.73 (0.42–1.26)	0.60 (0.34–1.04)	0.0016
		Leptin		
Abdominal obesity	1.00	1.28 (0.90–1.81)	1.96 (1.34–2.87)	0.0889
Elevated BP	1.00	1.41 (1.00–1.97)	1.55 (1.06–2.27)	0.0459
Elevated FBG	1.00	1.51 (1.05–2.16)	1.29 (0.85–1.95)	0.0316

Elevated TG	1.00	1.25 (0.90–1.74)	1.44 (0.99–2.08)	0.0163
Low HDL-C	1.00	1.13 (0.67–1.91)	0.98 (0.54–1.81)	0.0371
		LA ratio		
Abdominal obesity	1.00	1.39 (0.98–1.97)	1.85 (1.25–2.74)	0.0081
Elevated BP	1.00	1.30 (0.93–1.81)	1.50 (1.03–2.18)	0.1007
Elevated FBG	1.00	1.35 (0.94–1.94)	1.39 (0.92–2.09)	0.2104
Elevated TG	1.00	1.58 (1.11–2.24)	2.49 (1.71–3.65)	<0.0001
Low HDL-C	1.00	1.17 (0.71–1.92)	1.24 (0.67–2.31)	0.7637

MetS, metabolic syndrome; T, tertile; HR, hazard ratio; CI, confidence interval; BP, blood pressure; FBG, fasting blood glucose; TG, triglyceride; HDL-C, high-density lipoprotein cholesterol, LA ratio, leptin-adiponectin ratio; BMI, body mass index; hs-CRP, high-sensitivity C-reactive protein. [1] Model was adjusted for age (years), area of residence (Ansan or Ansung), education level (≤elementary school, middle/high school, or ≥college), smoking status (never, past, or current), alcohol consumption (g/day), regular physical activity (yes or no), BMI (kg/m^2), family history of diabetes (yes or no), and serum hs-CRP level (mg/L). [2] Tests for trend linearity were conducted with the Wald test by considering the median values of each tertile of adiponectin, leptin, and LA ratio as continuous variables in the analytical models.
